# COVID-19 Outbreak Forecasting Based on Vaccine Rates and Tweets Classification

**DOI:** 10.1155/2022/4535541

**Published:** 2022-10-27

**Authors:** Y. Didi, A. Walha, M. Ben Halima, A. Wali

**Affiliations:** ^1^Department of Computer Science, Umm Al-Qura University, Makkah 24243, Saudi Arabia; ^2^REsearch Groups in Intelligent Machines (REGIM-Lab), National Engineering School of Sfax, University of Sfax, Sfax 3038, Tunisia

## Abstract

The spread of COVID-19 has affected more than 200 countries and has caused serious public health concerns. The infected cases are on the increase despite the effectiveness of the vaccines. An efficient and quick surveillance system for COVID-19 can help healthcare decision-makers to contain the virus spread. In this study, we developed a novel framework using machine learning (ML) models capable of detecting COVID-19 accurately at an early stage. To estimate the risks, many models use social networking sites (SNSs) in tracking the disease outbreak. Twitter is one of the SNSs that is widely used to create an efficient resource for disease real-time analysis and can provide an early warning for health officials. We introduced a pipeline framework of outbreak prediction that incorporates a first-step hybrid method of word embedding for tweet classification. In the second step, we considered the classified tweets with external features such as vaccine rate associated with infected cases passed to machine learning algorithms for daily predictions. Thus, we applied different machine learning models such as the SVM, RF, and LR for classification and the LSTM, Prophet, and SVR for prediction. For the hybrid word embedding techniques, we applied TF-IDF, FastText, and Glove and a combination of the three features to enhance the classification. Furthermore, to improve the forecast performance, we incorporated vaccine data as input together with tweets and confirmed cases. The models' performance is more than 80% accurate, which shows the reliability of the proposed study.

## 1. Introduction

**Figure 1:**
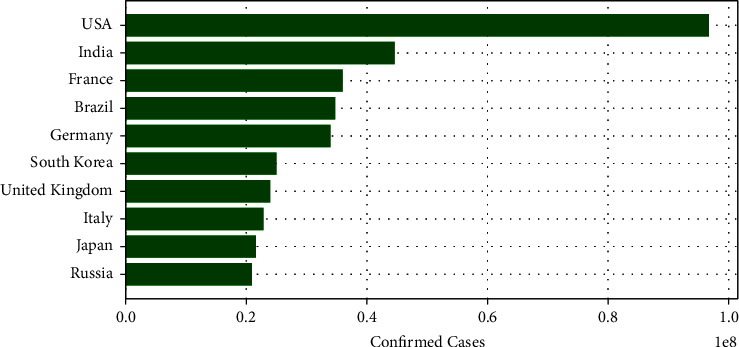
Top 10 countries of confirmed cases.

**Figure 2:**
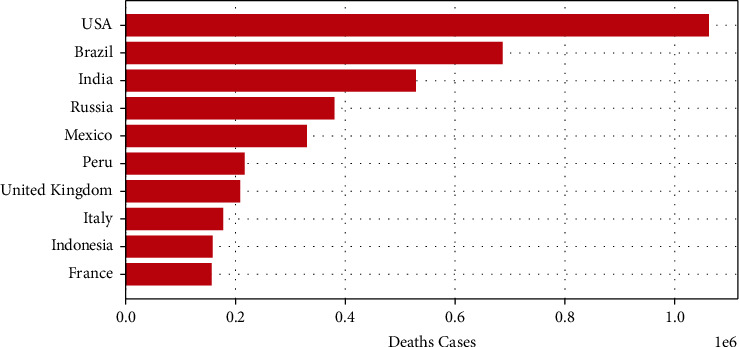
Top 10 countries of death cases.

The coronavirus disease is a worldwide pandemic that caused a critical threat to health systems. The deadly virus, first, was appeared in China in December 2019 and continues to pose problems to the economy and healthcare infrastructures worldwide [1]. As of February 2022, [2], the overall number of confirmed cases has surpassed 433,358,400, and the overall number of death cases exceeded 5,940,413 as shown in Figure 1 and Figure 2. Despite the effectiveness of the vaccine and the strict social distancing and preventive measures, even new viral strains have shown up in different countries such as Brazil, India, and the UK [3]. Many countries focused on urgent clinical decisions and efficient management of healthcare resources to reduce critical intervention. Meanwhile, many researchers focused on developing approaches and proposing solutions to generate earlier outbreak warnings.Recently, SNSs have been playing an indispensable role in disease surveillance. Twitter contains a huge amount of information about the disease through the experiences shared by the users when they express their opinions, news, and emotions facing the pandemic [4]. Researchers considered Twitter a valuable source for the detection and tracking of different events. Effective real-time screening and analysis of Twitter can assist the world in accurately predicting the disease pattern, figuring out the active cases at any given point in time, and being aware of the spread extent [5]. Extracting people's opinions is defined as the process of sentiment analysis (SA) by machine learning. It involves the analysis and classification of people's behavior toward the disease relying on three important categories: Positive, Neutral, and Negative. It is the most popular research in Natural Language Processing (NLP) [6, 7].

Supervised ML algorithms are considered techniques capable of solving many complex real-world problems and of designing a COVID-19 prediction model [8]. Several studies have applied machine learning algorithms to both classification [9–14] of social media tweets and forecasting [15–19] time series data to identify the rate at which the disease is spreading. Tweets-related COVID-19 classification is based on surveillance models that apply text processing and extracting knowledge from tweets related to the disease to generate earlier reports [20]. In most of the studies, tweets are classified into three sentiment types: positive, neutral, and negative using NLP and machine learning algorithms such as SVM, XgBoost, Random Forest, LR, LSTM, and CNN. These sentiments are very useful to build faster disease surveillance systems. Furthermore, the method to forecast COVID-19 time series is becoming at the heart of scientific research to track the pattern of confirmed and death cases and produce outbreak reports for the disease surveillance system. In addition, to establish a relationship between disease cases and people's opinions expressed on Twitter, a previous study proposed by [21] proved that the flu-related tweets collected during 2009 and 2010 are highly correlated with CDC reports of influenza like illness (ILI) cases.

Recently, ten vaccines have been developed to contain the pandemic. However, many inquiries have been raised about vaccines and their efficacy to decrease the fast growth of COVID-19. The study proposed by Xu et al., [22] on US data found that vaccines decrease non COVID-19 mortality. Also, Fukutani et al., [23] performed a study about the relationship between the daily vaccine rates and the infected cases. Their research found that 27 countries are negatively correlated to daily vaccination while 60 countries are positively correlated. These remarkable variations have raised questions related to the influence of vaccines on the growth of COVID-19 cases, which requires further investigations [24]. In addition, Almars et al., [25] studied the impact of vaccines on public opinions and health with the application of AI and IoT. The chapter presented an overview of the approaches and methodologies based on AI to predict the reaction and the uptake of the COVID-19 vaccine. Also, it discussed the measurements taken by governments to fight the spread of coronavirus.

Typical disease surveillance systems are based on reports of patients' visits provided by healthcare organizations to produce outbreak reports. Despite the accuracy of the official reports related to COVID-19 cases, the statistics are costly and take a long time to be issued. Therefore, a disease surveillance system needs to look for solutions to generate earlier outbreak warnings to prevent the disease's spread [26, 27]. The main purposes of this study are to find the best classification model and the best forecasting algorithm. First, the classification phase includes a pre processing step, feature extraction, and tweets classification. This step is based on feature extraction methods such as TF-IDF, Glove, and FastText which are very powerful word representations for machine learning classifiers. Initially, we combined the TF-IDF with FastText and TF-IDF with Glove to increase accuracy. Thereafter, these two different combinations were introduced in the classifiers for comparison purposes. Second, we compute the correlation between tweets, and the vaccine dataset with the confirmed and death cases to prove the relation and the effect of these factors on the disease outbreak. And finally, we integrated tweets and vaccine data to be used as estimators of the disease; it forecasts the COVID-19 trends relying on the newly introduced dataset (tweets, vaccination rate, and confirmed and death cases). The experimental results presented at the end show that our method produces a good forecasting performance.

The remaining of this paper is organized as follows: the literature body on the issue was introduced in section 2. The proposed approach was presented in section 3. Section 4, however, was devoted to the results analysis, and section 5 discussed the approach findings. Finally, the major conclusions of the paper were stated in section 6.

## 2. Related Works

COVID-19 surveillance systems have been examined by several data science researchers to analyze, understand, and track the patterns of the deadly disease. We, hereby, introduced some previous research on classification methods using different word representations: COVID-19-related classification works. Thereafter, many methods related to COVID-19 forecasting were introduced in COVID-19-related regression works.

### 2.1. COVID-19-Related Classification Works

A great deal of research that applied machine learning techniques to classify tweets using sentiment analysis has been achieved.

Initially, Gozes et al., [28] used an AI-based method for the detection and tracking of coronavirus on CT images which is able to distinguish coronavirus patients from non-patients. The proposed framework applied 2D and 3D deep learning algorithms combined with clinical reports. The method is able to evaluate the disease evolution in 157 patients from China and USA over time. The results achieved 98.2% of sensitivity and 92.2% of specificity. Recently, Samuel et al., [29] have developed a method that classifies the COVID-19 spread based on the Twitter dataset. They applied machine learning models such as logistic Naive Bayes (NB) and logistic regression (LR) to classify sentiment tweets into positive and negative classes. Their method performance was evaluated on the number of characters forming the data. The first data category counts 77 characters while the second had 120 characters per tweet. Their results revealed that NB outperformed LR in both data types. For tweets of 77 characters, the NB accuracy is 91.43% while LR achieved only 74.29%; as for the 120-character tweets, NB achieved 57.14%, whereas LR achieved 52%.

In the meantime, Imran et al., [30] applied the deep learning algorithm LSTM to classify sentiment-related COVID-19 tweets. They exploited the sentiment140 dataset with pre-trained Glove Twitter embedding as an input to the LSTM method. The main goal was to track sentiment polarity from users' emotions. They proved that there is a strong correlation between neighboring countries' polarity. Furthermore, in their publication, Alqurashi et al., [31] have explained the advantages of Word embedding methods to enhance the classification of machine learning models. They compared the performance of FastText, TF-IDF, and Word2vec word embedding techniques that are fed to several machine learning models such as RF, XgBoost, stochastic gradient descent (SGD), SVC, NB, and deep learning algorithms such as RNN, CNN, and CRNN. Their results show that FastText reached a high accuracy of 86.8% with XgBoost whereas Word2Vec performed a better accuracy with deep learning classifiers; it achieved 85.7% with CNN.

Moreover, Abdelminaam et al., [32] were able to identify the word features of COVID-19 misleading tweets. This was possible thanks to their optimization of the LSTM and GRU parameters with Keras-tuner to classify a large dataset of tweets into two categories: fake and nonfake. Also, they compared the performance of several machine learning algorithms such as LR, decision trees (DT), RF, K nearest neighbors (KNN), NB, and SVM. The dataset was transformed into an N-gram with TF-ID and was then passed to traditional classifiers. The dataset was represented with the word embedding method Glove for deep learning algorithms. Their experimental results prove that the modified LSTM outperformed GRU achieving 98.57% of accuracy. Noteworthy, the SVM bigram produced a better accuracy than the other models with 96.64%. Also, Priyantha et al., [33] developed an approach to identify the location of infected regions using the LSTM and SVM. The LSTM-based approach achieved better accuracy than the SVM. The comparative study developed by Wisesty et al., [34] was carried out by analyzing the tweet's sentiment using Kaggle competition data of three sentiment classes. They represented the tweets with Bag of Words and TF-IDF as input to the SVM, while Word2Vec and Glove as input to the LSTM and BERT. Their experiments show that BERT outperformed the other methods with a 0.85 weighted F1 score. Furthermore, Sitaula et al., [35] proposed an approach based on an ensemble of Convolutional Neural Networks to analyze Nepali tweets related to COVID-19. The tweets were represented by three types of feature extraction methods namely, FastText, domain-specific, and domain-agnostic to extract semantic information. The ensemble CNN proposed is to aggregate three different CNN methods that were applied to the three feature extraction methods separately to improve the classification process. The results show that the ensemble CNN achieved 68.7% of accuracy and outperformed traditional ML methods. Similarly, in the work presented by Sitaula et al., [36], a novel multi-channel CNN method was applied to Nepali COVID-19 tweets. For tweets representation and feature extraction, they applied four methods namely bag of words, domain-specific and FastText, and a combination of the three. The proposed MCNN model on the hybrid feature extraction method outperformed the traditional machine learning algorithms.

Much research focused on the effect of vaccines and people's reactions, we can cite among them the approach proposed by Okpala et al., [37]. They have developed a method to understand human perceptions towards the COVID-19 vaccine. Their approach is based on two machine learning algorithms: Naive Bayes and SVM, to analyze and classify the pro- and anti-vaccine tweets. The results obtained from their approach show that public opinions tend towards getting the vaccine. Lately, Akpatsa et al., [38] have studied and evaluated the public opinions of vaccine tweets. They applied different machine learning algorithms namely SVM, LR, RF, and NB to classify tweets on positive and negative reactions of people towards the COVID-19 vaccine. The experimental results show that SVM outperformed other algorithms with 84.32% of accuracy. Furthermore, Meyer [24] investigated the impact of COVID-19 vaccines on the mortality rate in Europe with machine learning algorithms. The study discussed the conflicting hypothesis either the increase of all-cause mortality is increased by the COVID-19 vaccines or the effect of vaccine decrease the non-COVID-19 mortality for different age categories. The results found that for a certain age group between 0 and 44 years, the benefit and risk balance does not promote the uptake of vaccines.

Several approaches have been applied and developed to analyze and investigate the emotions and reactions of people toward COVID-19 and the development of vaccines. However, hybrid approaches that combine different feature extraction methods can achieve better results and improve accuracy.

### 2.2. COVID-19-Related Regression Works

Numerous ML techniques have been developed in the literature to forecast the COVID-19 outbreak.

Initially, Chimmula and Zhang, [39] proposed an approach based on deep learning to predict future COVID-19 cases in Canada. They used the LSTM method on the coronavirus dataset to predict the pandemic pattern in the future. The prediction for the long-term period achieved 92.67% of accuracy while the short-term one reached 93.4%. Moreover, Bretschger et al., [40] presented a method that included environmental, economic, medical, and policy variables to analyze confirmed and deaths cases per million. Their empirical results proved that local air pollution has a positive impact on coronavirus infection and fatality rates.

A novel deep-learning algorithm has been employed by Shahid et al., [17]. They applied support vector regression (SVR), ARIMA, LSTM, and Bi-LSTM on a dataset gathered from Harvard University. The proposed approach analyses the rate of confirmed cases recovered and deaths in ten countries and they predicted the spread about 48 days ahead. The results proved that Bi-LSTM outperformed the other algorithms. Likewise, Prasanth et al., [41] developed a hybrid method on the Google Trends dataset. They applied the LSTM and ARIMA to forecast (future trends) using hyper-parameters improved by GWO (gray wolf optimizer). Their experiments showed that LSTM outperformed ARIMA. Furthermore, Farooq and Bazaz, [42] have developed a method that forecasts the pandemic in India using the artificial neural network (ANN). The model used the online incremental learning technique, in which its parameters were adapted intelligently to a new dataset, and was able to forecast the cases 30 days ahead in five badly-affected Indian states.

In the work proposed by Ballı [43], many machine learning methods like SVM, LR, RF, and multi-layer perception were implemented to study the disease pattern and forecast the epidemic curve. Their experiments show that SVM outperformed the other models. Further, the study proposed by Zain and Alturki, [44] presents a hybrid time-series method that combines CNN with LSTM to predict the number of infected cases. They compared the hybrid method with 17 machine learning models and evaluated on test and predicted data. The proposed approach achieved 13275 RMSE and 0.19 MAPE. Similarly, Ketu and Mishra [45] applied a hybrid CNN-LSTM to forecast the pandemic spread in India. The proposed hybrid deep learning model is divided into two parts. The first component is the use of pooling and consensual layers to produce the features of the input data and the second part is the use of LSTM and dense layers to employ the generated characteristics. Recently, Gupta et al., [46] have designed a method that uses Prophet, LR, and SVM to predict the infected, deaths, and active cases in India. The comparison of methods shows that Prophet achieved better results and it outperformed LR and SVM due to its characteristics to determine the growth curve and identify the change points in the dataset. Last but not least, in the data-driven model proposed by Alali et al., [47], several machine learning models were applied to forecast the confirmed and recovered cases in India and Brazil. To improve the prediction process they applied Bayesian optimization to optimize the Gaussian process regression (GPR) hyper-parameters. Also, they took into consideration the time dependency and lagged measurements in their study. The OGPR achieved superior performance compared to other models such as DT, XGBoost, RF, Bagged trees, Boosted trees, and SVR.

Despite the importance of existent research seen in the literature review, analyzing the pattern and growth of COVID-19 cases is still a prominent field for further studies, yet it could be interesting to study whether and how external factors such as vaccine rate and tweets can help in predicting and forecasting the COVID-19 confirmed cases.

## 3. Methods

**Figure 3:**
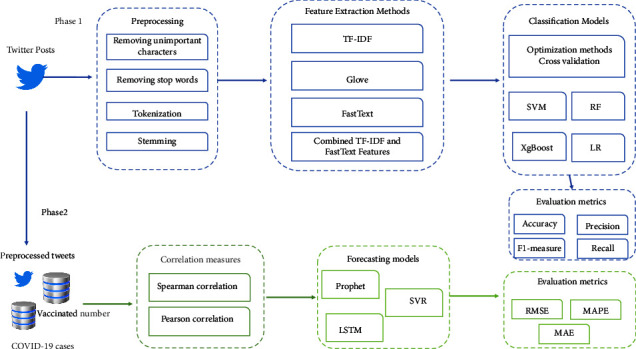
The proposed framework for COVID-19 forecasting.

In this section, we present the research design. Where it is divided into three steps: The first part focuses on collecting datasets related to COVID-19. The second part explains the process of classification and the last part dives into the forecasting phase.  Figure 3 illustrates the full proposed approach. Further details were discussed below.

### 3.1. Data Collection

Our experiments were conducted using three types of datasets including tweets, real-time coronavirus, and vaccine data.

**Figure 4:**
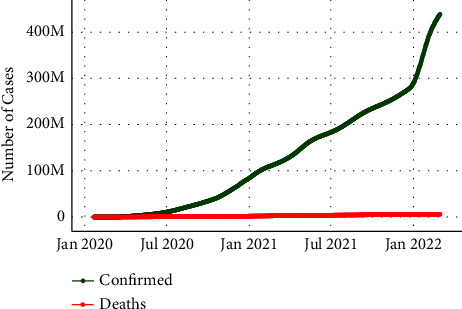
Worldwide COVID-19 outbreak trend.

**Figure 5:**
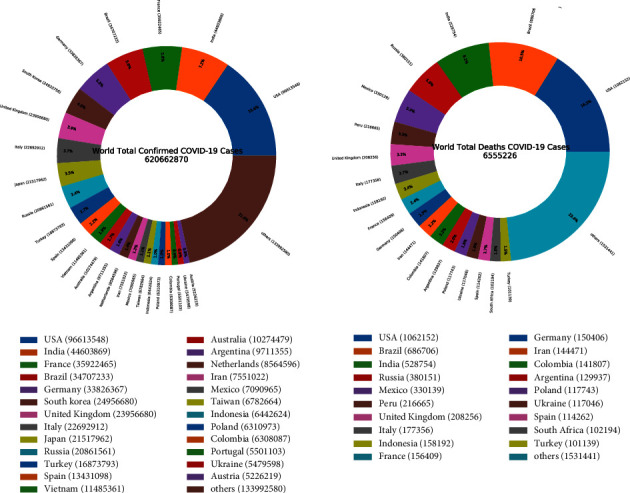
World Total confirmed and death cases till February 24, 2022.

**Figure 6:**
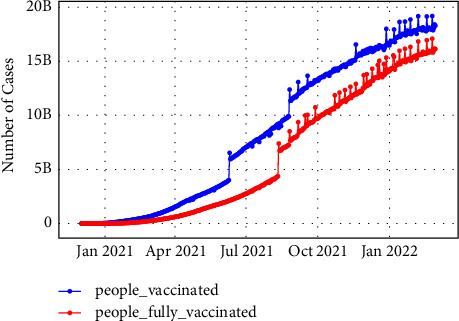
Worldwide COVID-19 vaccinated people.

1. Since Twitter policy does not allow tweet publication or access and streaming complete tweets to third parties, the first dataset used is about 470,394 tweets. It is freely available, continuously updated, and collected from IEEE port from 20^th^ March 2020 till 26^th^ February 2022. In this IEEE port [48], they used more than 90 keywords and hashtags to collect the tweet dataset. This data includes the tweet IDs and to capture the complete tweet information, we hydrated the dataset with the desktop application DocNow [49] which is a free open-source hydration software. The received information from DocNow is tweet IDs, tweet texts, location, creation time, and more. The tweets are in English language only in this study.
2. The second dataset includes the number of infection and death cases for each country worldwide and was extracted from an open-source freely available at the Johns Hopkins University Public Repository (COVID-19) [50]. The data repository is regularly updated and used as a dataset reference for comparison.  Figure 4 and Figure 5 illustrate the increase of confirmed and death cases till 24th February 2022 globally and per country.
3. The COVID-19 vaccination data used in this study were extracted from an open-source freely available GitHub [51] collected by Mathieu et al., [52]. The dataset contains the number of vaccinated people for each country from December 20^th^, 2020, as shown in Figure 6

## 4. Classification Phase

### 4.1. Tweets Data Pre Processing

**Figure 7:**
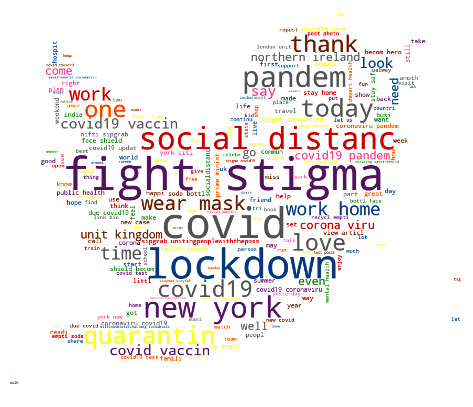
Word cloud of the dataset.

**Figure 8:**
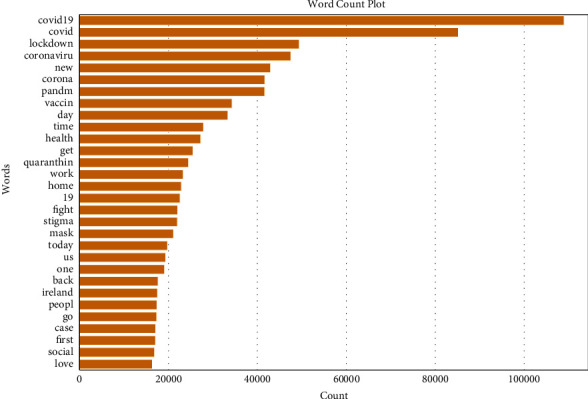
The most frequent word number.

The data pre processing phase is crucial for word processing as it impacts the performance of the sentiment systems. Usually, tweets involve different types of noises. They include text, URLs, special symbols, mentions, hashtags, emojis, and links which are not necessary for analysis. Thus, it is essential to clean the tweets before feeding them to machine learning classifiers by the application of natural processing techniques (NLP). These techniques involve several steps which are the removal of unimportant characters, stop-words, tokenization, and stemming; these are described as follows:

1. Remove an unimportant character: In this step, we removed emojis, special characters, irrelevant links, punctuation, and hashtags embedded into tweets using the NLP toolkit (NLTK) [53]. The stop-words also are removed as they are useless in the sentiment analysis. The text is converted into lower-case characters.
2. Tokenization: In this step, a sentence is split into a list of small pieces of strings called words or tokens with existing libraries in NLP [54].
3. Stemming: After tokenization, we applied the Porter stemming Algorithm [55], which is one of the most well-known stemming algorithms that help to reduce words to their roots.  Figure 7 illustrates the most significant words in the tweets with Word cloud representation and Figure 8 shows the most frequent words in the dataset.
4. Pre-trained Tweets: In this step, we applied the pre-trained models NLTK and TextBlob libraries [56] which usually have been trained on larger datasets to identify the sentiment polarity and subjectivity of each emotion or attitude of the Twitter writer. The people's reactions can be positive, negative, or neutral. Subjectivity refers to personal opinions whereas sentiment polarity identifies the orientation of the emotions to be positive, negative, or neutral. The value of polarity is between -1 and less than 0 refers to negative sentiment, 0 means the emotion is neutral whereas a score that is greater than 0 and less than 1 means the emotion is positively orientated. We used polarity to label the tweets.

### 4.2. Feature Extraction Techniques

The underlying idea of feature extraction is to project words in a continuous vector (or matrix) space of features. In this representation, probabilities are assigned to sentences and sequences. We chose to apply three of the most well-known feature extraction models on the historically COVID-19-related tweets, namely, Term Frequency-Inverse Document Frequency (TF-IDF), Glove, and FastText. The feature extraction process improves the degree of execution since raw tweets cannot be handled by machine learning algorithms.

#### 4.2.1. TF-IDF

The TF-IDF [57] is a very powerful and commonly weighted method used to extract features and evaluate word level in a document. We applied the TF-IDF N-gram on tweets where N can be one (uni-gram), two (bi-gram), three (tri-gram), and so on. It is performed in two methods, TF and IDF. TF refers to the total occurrences of term appearance in a document while IDF is the total frequency of all words in a given document. The product of TF with IDF is a weighted measure of word relevance. Equations (1) to (3) present the TF-IDF.

(1) $$\begin{array}{c} \text{TF}\left(t,d\right)=\frac{n_{t}}{n}, \end{array}$$

(2) $$\begin{array}{c} \text{IDF}\left(d\right)=\frac{N_{d}}{N}, \end{array}$$

(3) $$\begin{array}{c} \text{TF}-\text{IDF}\left(t,d\right)=\text{TF}\left(t,d\right)\times \text{IDF}\left(t\right), \end{array}$$

where *t* refers to the word with frequency *n*, *d* represents the document and *N* refers to the frequency *d* of a text containing the term *t*.

#### 4.2.2. Glove

The glove is a Global Vector for Word Representation of the Stanford NLP group [58]. This embedding word vector is mostly used for feature extraction and the creation of a dictionary of keywords and their corresponding list of values. A feature matrix is generated based on feature-feature co-occurrence that matches each row with a word index. In this study, we applied pre-trained word vectors from freely available corpora with 6 billion tokens from Common Crawl.

#### 4.2.3. FastText

FastText [59] is a word-embedding approach provided by a Facebook team based on the skip-gram model. Each word in the corpus is transformed into an N-grams character and associated with the sum of the vector representation of each N-gram character and even rare or misspelled words will have an embedding matrix. In this study, each raw tweet was converted to an embedding matrix with the help of a pre-trained model based on 1-million-word vectors trained on Wikipedia 2017 with 1 billion tokens.

### 4.3. Hybrid Feature Extraction Techniques

Hybrid feature extraction techniques were proposed to improve the classification models and get valuable knowledge and learning. To this end, our suggested model combined the recent embedding word models TF-IDF with FastText and TF-IDF with Glove to enhance the machine learning algorithms' performance. The representation of the tweets syntactically can be improved with the semantic features since the two methods are complementary and efficient in word representation. The context information was performed by the FastText-based method and Glove-based method whereas the syntactic information was introduced by the TF-IDF.

In this paper, we made a series of comparisons of feature extraction techniques on Twitter data with different machine-learning models. The two combination sets take advantage of their complementarity and resulted in an improvement of the different models. 300-dimensional vectors were used to represent each word. The (4) for matrices fusion of both word representations is expressed as follows:

(4) $$\begin{array}{c} S_{ij}=\overset{M}{\underset{k=1}{\sum }}x_{ik}y_{kj}, \end{array}$$

where *M* is the number of tokens, *x* and *y* are the syntactic and semantic matrices, respectively, and *S* represents the final fusion matrix.

### 4.4. Machine Learning Models

In this paper, we proposed four machine learning algorithms, namely the SVM, XgBoost LR, and RF, to classify tweets into positive, neutral, and negative.

- Support Vector Machine: SVM is a classification-supervised machine learning model [60]. The features are projected in n-dimensional spaces providing coordinates to decide the value of an item. The categories are divided by a hyper-plane to achieve classification. Classification is achieved by discovering the best dividing hyper-plane of the categories. The SVM has different function types such as Gaussian/radial or kernel (linear, polynomial), we used the kernel function in this research.
- XgBoost: is the abbreviation of Extreme Gradient Boosting proposed by Tianqi Chen. The processing time and memory space have been optimized and were noticed to be very fast compared to the other boosting algorithms. The main idea of XgBoost is that the predictive value is chosen randomly after computing the average value of one feature. XgBoost [61] learns from the previous error made by the model and improves its next performance.
- Random Forst: RF is a supervised machine learning algorithm that belongs to the ensemble bagging techniques [62]. It contains a number of decision trees, instead of one, each of which was trained independently to improve accuracy, resulting in an enhancement of the RF performance. The main object was to learn from the feature decision rule to classify the target value variable of data provided by more than one tree.
- Logistic Regression: LR is a popular machine learning algorithm developed by [63], basically used to classify categorical sentiment with probabilities.

## 5. Prediction Phase

The ongoing COVID-19 pandemic has to be analyzed and diagnosed accurately to ensure the best prediction results possible. To predict the COVID-19 cases curve, we used a regression estimator. The proposed model implements different regression algorithms such as LSTM, Prophet, and SVR. The dataset used is the historical number of cases, the number of vaccinated people, and the COVID-19-related tweets collected from the classification phase are considered as external factors to the regression model. From the large variety of existing studies, we can conclude that the developed methods have advantages and limitations depending on certain situations. We suggest applying machine learning algorithms, such as SVR, Prophet, and deep learning models like LSTM, to efficiently predict and analyze the COVID-19 cases outbreak. In order to improve the prediction phase, we integrated the external indicators to help the models increase their accuracy. As indicators, we chose the number of vaccinated people and the COVID-19-related tweets obtained from the classification results as they show a high correlation (as seen in dataset correlation) with COVID-19 cases. The following step was to compare the models to find out the most accurate. First, we applied each model without any external indicators; then, we analyzed and studied the real influence of these variables on the pandemic spread.

### 5.1. Dataset Correlation

In the second phase of our surveillance system, we integrated some external indicators to enhance the prediction algorithms' performance. The indicators are the number of vaccinated people and COVID-19 and the related tweets obtained from the classification results. The correlation between the different resources was performed using the Spearman and Pearson correlation. The parameter *r* is the metric generated by these two methods. Its value varies between 1 and -1: If *r* tends to 1 or -1, the two datasets are strongly related. However, when *r* equals 0, there is no correlation between the two datasets.

Twitter data is used by many studies to build faster COVID-19 surveillance systems.  Table 1 shows the relationship between tweets and COVID-19 cases/deaths. The relation between the dependent variable (number of infections) and the independent variable (Tweets) was evaluated using Pearson correlation. As shown in Table 1 we can observe that *r* achieved a 64.1% of Pearson correlation between the two datasets. This positive correlation means that the two dataset increase in the same direction. Thus, we can integrate the trend of tweets in the process of prediction to enhance the accuracy of the time series prediction. It is a powerful indicator of disease control.

Recently, several vaccines have been developed to contain the pandemic. However, new cases have been increasing throughout the world, which raises certain questions about the efficiency of the vaccine in reducing this spread. A study by Fukutani et al., [23] proposed a comparison analysis between the daily vaccinations and their influence on COVID-19 spread. The authors found that there are 27 countries where coronavirus cases are negatively correlated with daily vaccination showing the great success of vaccination. 60 countries, however, are positively correlated. In fact, despite the vaccination process, COVID-19 cases are on the increase in certain countries.  Table 2 shows the correlation analysis between daily vaccination and cases/deaths in some countries. As observed in this table, the external variable has a strong influence on the pandemic pattern. Thus, it can be concluded that this correlation could be a robust parameter of disease surveillance. Monitoring the correlation patterns can help countries to track the immunization program thanks to the vaccination system.

### 5.2. Machine Learning Models

We used the algorithms LSTM, Prophet and SVR in the second phase to forecast the infection and death cases and provide a complete analysis of the COVID-19 pattern.

#### 5.2.1. Prophet

Prophet is an open-source algorithm for time series analysis and forecast. It was designed by a Facebook team [64] primarily to forecast Facebook business and it is available in two languages: Python and R. The basic implementation of Prophet is to fit trends seasonality that is non-linear. Equation. (5) of the Prophet is expressed as follows:

(5) $$\begin{array}{c} k\left(t\right)=\text{tr}\left(t\right)+\text{set}+\text{ho}\left(t\right)+\text{id}\left(t\right), \end{array}$$

where tr denotes the trend and non-periodic changes, se is the seasonality or the periodic changes, ho defines the effect of holidays and id is the individual changes. The major strength of Prophet is its ability to deal with missing values and detect anomalies. In Prophet, the prediction model consists of two basic columns. The first column “ds” is for date storage. The “y” column is to store the independent time series data. Therefore, the method can efficiently be applied and handled for seasonal time series dataset.

#### 5.2.2. Long Short-Term Memory

**Figure 9:**
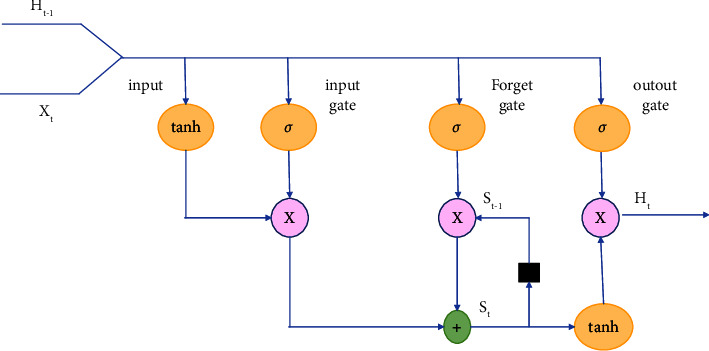
Lstm architecture.

LSTM is deep learning model based on recurrent neural network. It was developed by Hochreiter and Schmidhuber, [65] in 1997 to overcome the RNN inability to memorize long term dependencies due to memory shortage. The LSTM main characteristic is its ability to memorize data for long periods of time in the hidden layers. The LSTM architecture consists of memory units, i.e. memory cells, and gate units for input, output and forget gate with an activation function which are needed to store and use background information (see Figure 9).

The memory blocks are implemented as shown in the following equations:

(6) $$\begin{array}{c} I_{t}=\sigma_{g}\left(W_{1}X_{t}+U_{i}C_{t-1}+b_{1}\right),\\ F_{t}=\sigma_{g}\left(W_{2}X_{t}+U_{f}C_{t-1}+b_{2}\right),\\ O_{t}=\sigma_{g}\left(W_{3}X_{t}+U_{o}C_{t-1}+b_{3}\right),\\ C_{t}=F_{t}⊙C_{t-1}+I_{t}⊙\sigma_{c}\left(W_{c}X_{t}+b_{4}\right),\\ H_{t}=O_{t}⊙\sigma_{h}\left(C_{t}\right). \end{array}$$

The equations explained above include the variables *W* and *U* which represent the weights between 0 and 1, the vector *F*_*t*_ represent the forget gate while *X*_*t*_, *O*_*t*_, *H*_*t*_ and *b* represent the input vector, the output gate vector, the LSTM units output vector, and the bias vectors, respectively.The operator ⊙ denotes the Hadamard product, [65, 66]. The evaluation criteria are measured by a cost function of the trained LSTM cell with the back propagation algorithm. The used algorithm computes the flu activity *O*_*t*−*i*_ of data at time *t* − *i* received from previous LSTM cell *O*_*t*−*i*−1_ and the input *X*_*t*−*i*_.

#### 5.2.3. Support Vector Regression

SVR uses the same basis idea as SVM but it is applied for regression models to forecast real values and to solve non-linear problems with few samples. Not only knowing by its efficiency to solve high dimensional problems but also it is able to extract correlations between output and input data.

## 6. Experimental Results

### 6.1. Performance Metrics

For the classification phase, we adopted four evaluation metrics including accuracy, precision, recall and F-measure. We also referred to two evaluation metrics for forecasting which are the RMSE, MAP, and MAE.

(i) Accuracy indicates the mean of both recall and precision and its equation is written as follows:
  (7) $$\begin{array}{c} \text{Accuracy}=\frac{\text{TP}+\text{TN}}{\text{TP}+\text{TN}+\text{FP}+\text{FN}}. \end{array}$$
(ii) Precision score represents the percentage of positively classified tweets that are actually correct. Precision is mathematically expressed as follows:
  (8) $$\begin{array}{c} \text{Precision}=\frac{\text{TP}}{\text{TP}+\text{FP}}. \end{array}$$
(iii) Recall score indicates the ability of the classifiers to classify all positive instances correctly. Recall is mathematically expressed as follows:

(9) $$\begin{array}{c} \text{Recall}=\frac{\text{TP}}{\text{TP}+\text{FN}}, \end{array}$$

where TP refers to True Positives, the number of correctly predicted instances of the positive class; TN is True Negatives, the number of correctly predicted instances of the negative class; FP refers to False Positives, the number of incorrect positive predictions of a class; FN refers to False Negatives, the number of incorrect negative predictions of a class.

(i) RMSE: root mean square error is the metric used to measure the difference between the predicted value and the actual one.
  (10) $$\begin{array}{c} \text{RMSE}=\sqrt{\frac{1}{N}\Sigma \frac{{\left(A-P\right)}^{2}}{A}}\times 100. \end{array}$$
(ii) MAPE: mean absolute percentage error is the metric used to measure the accuracy in percentage: it is the average absolute error between actual and estimated values.
  (11) $$\begin{array}{c} \text{MAPE}=\frac{1}{N}\Sigma \frac{\left|A-P\right|}{\left|A\right|}\times 100. \end{array}$$
(iii) MAE: mean absolute error is the metric used to measure the average absolute error between actual and estimated values.

(12) $$\begin{array}{c} \text{MAE}=\frac{1}{N}\Sigma \left|A-P\right|. \end{array}$$

### 6.2. Classification Performance

The word embedding methods performance was evaluated on several machine learning algorithms for classification. First, the Synthetic Minority Oversample Technique (SMOTE) algorithm proposed by Farquad and Bose [67] was applied on the tweet dataset to avoid the bias of imbalanced samples. The tweets of COVID-19 were divided into training and testing sets with the ratio of 80 : 20. We obtained 376,315 tweets for the training and 94,079 for testing.

The combination of syntactic and semantic features of the proposal model improves the performance of COVID-19 tweets classification. In this method, we relied on Glove and FastText which can yield the average weighted pooling.  Table 3 displays the achieved performance comparison results. These results were obtained using the three different features together with the two proposed combinations. Then, we applied the four classifiers, namely LG, XGBoost, RF and SVM using the Accuracy, Precision, Recall, and F-measure metrics to classify the tweets into three categories: positive, neutral, and negative.

**Figure 10:**
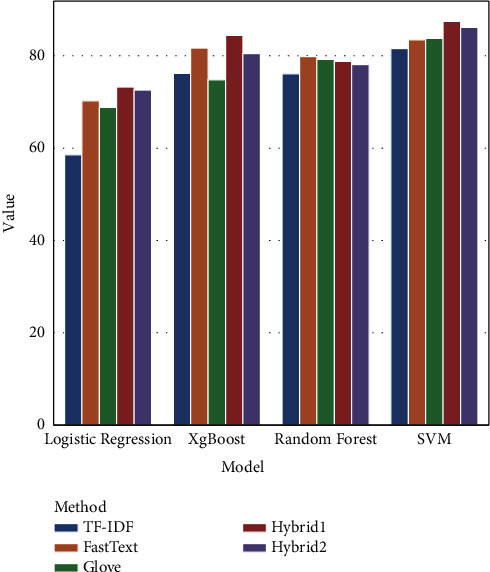
Performance Accuracy of four machine learning classifiers with features extraction.

Table 3 and Figure 10 show that the machine learning models performed differently with the different features. The SVM achieved the highest accuracy on TF-IDF (81.58%), FastText (83.48%), Glove (83.76%), Hybrid 1 (87.5%) and Hybrid 2 (86.15%), respectively. Likewise, XGBoost achieved fairly good results with the first approach where accuracy was 76.20% on TF-IDF, 81.66% on FastText, and 74.80% on Glove. Its implementation a better achievement than FastText by 2.77% reaching 84.43% and by 5.66% on TF-IDF based FastText reaching 80.46% on TF-IDF based Glove; but it is still less accurate than FastText alone. Similarly, relying on LR, the performance was improved by 2.99% compared to FastText, achieving 73.24% on TF- IDF based FastText and by 3.74% on TF-IDF based Glove (72.56%). On the contrary, RF did not improve its performance with the different metrics. In fact, when we combined both FastText or Glove with TF-IDF, the accuracy dropped. The RF execution decreased by 1.03% compared to FastText (79.83%) and by 1.13% compared to Glove (79.23%). We can deduce that the combination of different features to classify sentiments achieved better results than the three word embedding methods with the different metrics: Accuracy, Precision, Recall and F-measure for almost all the ML methods except for RF. Furthermore, the hybrid TF- IDF based FastText outperformed the hybrid TF- IDF based Glove in most algorithms thanks to the ability of FastText to capture the contextual information and to go along with TF-IDF in representing tweets accurately. From the achieved result, we can conclude that when the syntactic representation was used in combination with the semantic representation, the results improved showing its complementarity and its novel contribution to the method.

### 6.3. Regression Performance

The main purpose of this phase was to use the available data and methods to accurately and effectively predict the evolution of COVID-19 over time in the world. To this end, different predictors such as tweets and vaccine rate as independent variables were used to predict the COVID-19 dataset as a dependent variable. Based on the correlation presented in Dataset Correlation, we evaluated the relationship between the independent and dependent variables. We noticed that each variable affected predictions with a varying amount of time lag. In the learning process, the models decide what relevant time windows is for each variable to future predictions. The tweets and vaccine rate have a seven-day delayed effect on COVID-19 cases. Thus, the variables were lagged to improve the model efficiency for COVID-19 cases.

The performances of three different models LSTM, Prophet, and SVR were compared to RMSE, MAPE, and MAE. We trained these models with the ratio of 80 : 20 for training and testing data.  Table 4 displays the results for each evaluation metric. The multivariate LSTM outperformed the multivariate Prophet and SVR. The multivariate LSTM achieved 310.48 of RMSE, 100% of MAPE, and 173.7 of MAE while Prophet achieved 570.63 of RMSE, 120% of MAPE, and 206.05 of MAE. Furthermore multivariate SVR achieved better results than univariate SVR with 642.58 of RMSE, 0.19 of MAPE, and 316.45 of MAE. We further compared the proposed approach against the univariate LSTM, Prophet, and SVR. From Table 4, we can observe that these models achieved high RMSE, MAPE, and MAE; they are less accurate in forecasting the confirmed cases.

The obtained results show that including the external factors enabled less RMSE, MAPE, and MAE, which allows us to confirm the importance of including other possible and correlated factors to enhance the performance of both LSTM, Prophet, and SVR models.

**Figure 11:**
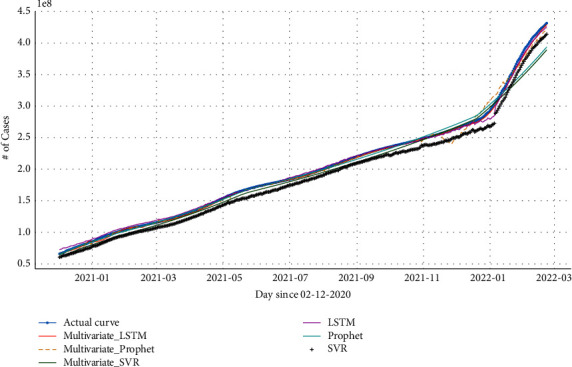
Forecast results of Global accumulating cases using LSTM vs. Prophet vs. SVR.

Moreover, the experimental results curve and the actual growth of the number of cases for each model for the world are shown in Figure 11 with and without external variables. For illustration purposes, we display the data from December 02^nd^, 2021, the starting day of vaccination.

## 7. Discussion

In this study, two main processes were performed: COVID-19-related tweet classification and COVID-19 forecasting. The tweet classification used several word embedding features to analyze sentiment. The word embedding evaluation shows that building an effective representation of tweet information is a crucial objective for researchers. The achieved results are interesting because of the relation between syntactic features and semantic information where the fusion helped to improve accuracy, precision, recall, and F-measure for several supervised classifiers.

Our second step was the forecasting process where we used the output data from the previous step as an independent factor together with vaccination aiming to improve COVID-19 prediction. These two external factors were incorporated together with the newly-confirmed cases, were chosen because of their relation and effects on the disease curve. They were correlated and evaluated relying on Pearson correlation metrics. We compared different types of machine learning algorithms like the LSTM, Prophet and SVR with different metrics. Additionally, we evaluated our proposed approach with Pearson correlation metric *r* value. From Table 5, *r* value is equal to 0.98 for the LSTM multivariate, 0.97 for the Prophet multivariate and 0.952 for multivariate SVR. This value proves the strong correlation between the proposed approach and the actual curve.

For further evaluation, Table 6 compares the performance of the proposed model with the state-of-the-art methods with the evaluation metrics. We notice that our approach outperformed the other methods with less RMSE and MAE values. Seeing that incorporating external factors in relation and correlated with COVID-19 data may help in forecasting COVID-19 outbreak and may observe the dynamics spread of any other disease.

## 8. Additional Points

While the results of our proposed study have achieved high accuracy, we admit that there are some challenges have to be addressed that my further enhance the performance of COVID-19 prediction overall. First, the study has only gathered tweets in English language despite the existence of rich Twitter datasets in other language such as Arabic. Secondly, we did not investigate and combine other feature extraction methods such as Bag of Words, Bert or domain-specific, and domain-agnostic proposed by [35]. Even though we applied several traditional classifiers, we did not study the performance of deep learning methods on the tweets. Finally, some other external factors may help the process of forecasting such as environmental factors, healthcare measures, socioeconomic, demographic variables, and more.

## 9. Conclusions

During COVID-19 outbreaks, several approaches have been developed to monitor the disease for early warning. Two branches, classification and forecasting, were explored and studied using several machine learning algorithms and data resources. SNSs data such as Twitter is a huge data source for disease prediction. To build an effective COVID-19 surveillance model, we suggested two hybrid pipeline methods; first we combined two word-embedding methods to better represent the tweet information syntactically and semantically with TF-IDF, FastText, and Glove. This hybrid method helped to improve the classification supervised algorithms. Thereafter, we incorporated external factors that can assist to visualize and estimate the changes of COVID-19. A combination of the classified tweets and vaccine rate were also integrated to the forecasting LSTM, Prophet, and SVR models. The experimental results showed the accuracy and performance of the proposed approach. In fact, the model achieved 98.76% of Pearson correlation with the actual confirmed cases.

As a future perspective, we consider examining and analyzing other external factors that can impact the forecasting process such as lockdown measures, age, population size, and density to enhance the COVID-19 prediction and better fight the pandemic.

## Figures and Tables

**Table 1:** Spearman Correlation between Tweets and COVID-19 cases.

	Confirmed	Deaths
R-value	64.1%	61.1%
*P*-value	1.202635e−51	7.893323e−47

**Table 2:** Correlation between the numbers of vaccines and COVID-19 cases.

	Confirmed		Deaths	
	R-value	*P*-value	R-value	*P*-value
India	0.881	1.28E−32	0.681	1.60E−14
Tunisia	0.829	3.90E−11	0.796	8.38E−10
UK	−0.557	3.83E−10	−0.521	7.28E−09
USA	−0.78	1.94E−26	−0.774	8.82E−26
Saudi Arabia	0.78	4.51E−18	0.767	8.35E−22

**Table 3:** Performance of traditional classifiers on features extraction.

	Metrics	LR	XGBoost	RF	SVM
TF-IDF	Accuracy	58.58%	76.20%	76%	81.58%
	Precision	0.59	0.76	0.76	0.81
	Recall	0.59	0.76	0.76	0.81
	F1-score	0.59	0.76	0.76	0.81

FastText	Accuracy	70.25%	81.66%	79.83%	83.48%
	Precision	0.70	0.82	0.80	0.83
	Recall	0.70	0.82	0.80	0.83
	F1-score	0.70	0.82	0.80	0.83

Glove	Accuracy	68.82%	74.80%	79.23%	83.76%
	Precision	0.69	0.75	0.79	0.84
	Recall	0.69	0.75	0.79	0.84
	F1-score	0.69	0.75	0.79	0.84

Hybrid 1^1^	Accuracy	73.24%	84.43%	78.80%	**87.5%**
	Precision	0.73	0.84	0.79	**0.87**
	Recall	0.73	0.84	0.79	**0.87**
	F1-score	0.73	0.84	0.79	**0.87**

Hybrid 2^2^	Accuracy	72.56%	80.46%	78.10%	**86.15%**
	Precision	0.73	0.80	0.78	**0.86**
	Recall	0.73	0.80	0.78	**0.86**
	F1-score	0.73	0.80	0.78	**0.86**

^1^Hybrid TF-IDF with FastText. ^2^Hybrid TF-IDF with Glove. The meaning of the bold values is only to highlight the high value achieved by each technique.

**Table 4:** Performance of COVID-19 using different regression models.

ML models	RMSE	MAPE	MAE
LSTM	869.39	4.40	381.96
Prophet	708.09	1.34	656.05
SVR	750.32	2.25	434.5
LSTM + tweet + vaccine	310.18	1	173.7
Prophet + tweet + vaccine	570.63	1.2	206.05
SVR + tweet + vaccine	642.58	0.19	316.45

**Table 5:** Evaluation of Pearson correlation for different models.

Models	*r*-value
LSTM	0.969
Prophet	0.957
SVR	0.930
LSTM + tweet + vaccine	0.988
Prophet + tweet + vaccine	0.974
SVR + tweet + vaccine	0.952

**Table 6:** Performance comparison against the state-of-the-art methods.

References	Models	RMSE	MAPE	MAE
Zain and alturki [44]	Hybrid CNN-LSTM	12366.00	0.54	560.2
Ketu and mishra [45]	Hybrid CNN-LSTM	880.490	21.00	259.3
Alali et al. [47]	Optimized Gaussian process regression	130714.951	0.465	119842.670
Our method	LSTM + tweet + vaccine	310.180	1	173.7
Our method	Prophet + tweet + vaccine	570.630	1.2	206.05

## Data Availability

The first dataset used to support the findings of this study is available on IEEE at http://ieee-dataport.org/open-access/coronavirus-covid-19-geo-tagged-tweets-dataset(accessed on 24 February 2022). The second dataset used is available on https://github.com/CSSEGISandData/COVID-19 (accessed on 24 February 2022) and the third dataset used is available on https://github.com/owid/covid-19-data/tree/master/public/data/vaccinations (accessed on 24 February 2022).
